# Evaluation of the endotoxin binding efficiency of clay minerals using the *Limulus Amebocyte* lysate test: an *in vitro* study

**DOI:** 10.1186/2191-0855-4-1

**Published:** 2014-01-02

**Authors:** Simone Schaumberger, Andrea Ladinig, Nicole Reisinger, Mathias Ritzmann, Gerd Schatzmayr

**Affiliations:** 1 BIOMIN Research Center, Technopark 1, Tulln 3430, Austria; 2 Clinic for Swine, University of Veterinary Medicine Vienna, Veterinaerplatz 1, Wien 1210, Austria; 3 Clinic for Swine, Ludwig-Maximilians-University Munich, Sonnenstrasse 16, Oberschleissheim 85764, Germany

**Keywords:** Endotoxin, Lipopolysaccharide, Bentonite, Organoclay, Binding, Isotherm, LAL test, Feed additive

## Abstract

Endotoxins are part of the cell wall of Gram-negative bacteria. They are potent immune stimulators and can lead to death if present in high concentrations. Feed additives, which bind endotoxins in the gastrointestinal tract of animals, could help to prevent their negative impact. The objective of our study was to determine the potential of a bentonite (Bentonite 1), a sodium bentonite (Bentonite 2), a chemically treated smectite (Organoclay 1) and a modified attapulgite (Organoclay 2) to bind endotoxins *in vitro*. Polymyxin B served as positive control. The kinetic chromogenic *Limulus Amebocyte* lysate test was adapted to measure endotoxin activity. Firstly, a single sorption experiment (10 endotoxin units/mL (EU/mL)) was performed. Polymyxin B and organoclays showed 100% binding efficiency. Secondly, the adsorption efficiency of sorbents in aqueous solution with increasing endotoxin concentrations (2,450 – 51,700 EU/mL) was investigated. Organoclay 1 (0.1%) showed a good binding efficiency in aqueous solution (average 81%), whereas Bentonite 1 (0.1%) obtained a lower binding efficiency (21-54%). The following absorbent capacities were calculated in highest endotoxin concentration: 5.59 mg/g (Organoclay 1) > 3.97 mg/g (Polymyxin B) > 2.58mg/g (Organoclay 2) > 1.55 mg/g (Bentonite 1) > 1.23 mg/g (Bentonite 2). Thirdly, a sorption experiment in artificial intestinal fluid was conducted. Especially for organoclays, which are known to be unspecific adsorbents, the endotoxin binding capacity was significantly reduced. In contrast, Bentonite 1 showed comparable results in artificial intestinal fluid and aqueous solution. Based on the results of this *in vitro* study, the effect of promising clay minerals will be investigated in *in vivo* trials.

## Introduction

Endotoxins are toxins that are kept in the cell wall of Gram-negative bacteria. In pure chemical form endotoxins are so called Lipopolysaccharides (LPS). LPS consist of a lipid part (lipid A) and a polysaccharide part with an inner core and O-specific side chains. They are characterised as amphiphilic (hydrophilic and lipophilic) molecules (Hodgson 2006), which are heat resistant and very pH stable. Endotoxin activity is indicated as endotoxin units (EU). In general, around 10 EU are equivalent to 1 ng endotoxin.

Being part of bacteria, endotoxins are continuously released into the environment (Magalhaes *et al.*^2007^). In the gastrointestinal tract, endotoxins are potent immune stimulators (Mani *et al.*^2012^), which in healthy animals are removed from the gut via the reticuloendothelial system (Munford 2005). If there is a failure in endotoxin deactivation, e.g. in cases of stress in animals, circulating endotoxin can lead to systemic inflammation, endotoxemia, shock and even death (Mani *et al.*^2012^; Rice *et al.*^2003^; Zweifach and Janoff 1965).

A feed additive**,** which binds endotoxins in the gastrointestinal tract, especially in a state of overwhelming release of endotoxins and in a state of a suppressed immune system, could help to inhibit the negative impact in the animal. The idea of feeding various adsorbents (e.g. bentonite, organoclays, kaopectate, charcoal, kaolin, terra fullonica, smectite) to endotoxin or *E. coli* challenged animals for prevention of related diseases (especially endotoxemia), has been investigated regularly (Ditter *et al.*^1983^; Gardiner *et al.*^1993^; Song *et al.*^2011^; Trckova *et al.*^2004^). However, none of the investigated adsorbents could be clearly assigned to endotoxin binding.

Besides the *in vivo* approaches, the efficacy of different substances to remove endotoxins from blood (dialysis), protein solutions and pharmaceuticals has been tested extensively. Different methods (e.g. ion-exchange chromatography, ultrafiltration, sucrose gradient centrifugation) and materials (e.g. L-histidine, poly-L-lysine, polymyxin B) have been known since the early 80′s and were described by Tosa *et al.*( 1987), Petsch and Anspach ( 2000) and later reviewed by Magalhaes *et al.*( 2007).

Currently, the most sensitive test for detecting endotoxins is the kinetic chromogenic *Limulus Amebocyte* lysate (LAL) test. The principle of the LAL test, discovered by Bang ( 1956), is that a lysate prepared from the blood of *Limulus polyphemus*, the horseshoe crab, forms clots when endotoxins are present (Young *et al.*^1972^). Coagulation is caused by the activation of a number of enzymes located in the blood cells (amebocytes) of *Limulus polyphemus*, and this activation is initiated by endotoxins. In the chromogenic test, the coagulogen has been replaced by a chromogenic substrate. The last enzyme activated in the cascade splits the chromophore from the chromogenic substrate, producing a yellow colour reaction proportional to the amount of endotoxins in the system in a time dependant manner (Hurley 1995). The biological reagent and the high sensitivity (0.005 endotoxin units/mL) of the test imply susceptibility for interferences (Dubczak 2011). These interferences have to be overcome with sample preparation and dilution finding for each matrix.

Although testing of potential endotoxin binding materials, like bone char (a form of charcoal produced by heating bone in the presence of a limited amount of air), using the LAL test has been reported (Rezaee *et al.*^2009^), there is still not much data available on promising protocols for the kinetic chromogenic LAL assay for the evaluation of clays like bentonites and organoclays as potent endotoxin sorbents.

The objective of our study was to determine *in vitro* the potential of bentonites and organoclays for an application as feed additive to prevent animals from endotoxin triggered diseases. The LAL test was adapted to evaluate endotoxin binding in aqueous solutions and artificial intestinal fluid. Results of our study helped us to determine the binding efficiencies of the tested materials.

## Material and methods

### Chemicals and reagents

Lipopolysaccharide (LPS) from *E. coli* O55:B5 (≥ 500,000 EU/mg) was purchased from Sigma-Aldrich Co. LLC (Vienna, AT). Polymyxin B- sulfate (PMB), an antibiotic, which is known to bind and inactivate endotoxins, was obtained from AppliChem (Darmstadt, D). Two bentonites (Bentonite 1 – main part smektite), one being a natural sodium-bentonite (Bentonite 2 – main part consist of smectite, feldspar and gypsum), and two clays treated with amines (organophilised) were used: a smectite (Organoclay 1 – fully organophilised: whole surface coated with amines) consisting of montmorillonite and an attapulgite (magnesium phylosillicate) consisting of smectite and palygorskite (Organoclay 2 – part of the surface organophilised).

Reagents used for the *Limulus amebocyte* lysate (LAL) test were obtained from Charles River Laboratories (CRIVER), Inc. Charleston, US: *Limulus amebocyte* lysate (Endochrome K; Charge: C4452E), Endotoxin-free (< 0.005 EU/mL) LAL reagent water (LRW; Charge: 99732088) and Endosafe control Standard Endotoxin from *E. coli* O55:B5 1.000 EU/mL (Charge: EX 14392 and EM11302 – RSE/CSE ratio 10 EU/ng; EX01022 – RSE/CSE ratio 12 EU/ng; EM11302 – RSE/CSE ratio 7 EU/ng). Potassium phosphate monobasic, sodium hydroxide and pancreatin for the preparation of the artificial intestinal fluid were obtained from Merck KGaA (Darmstadt, D).

### Materials

All materials used in the experiments were pyrogen free. Glass tubes were obtained from ACILA (16 × 90 mm PYROKONTROL® tubes capped; Weiterstadt, GERD). Material which was reused was heat depyrogenated for 3 h at 230°C. 1.5 mL reaction tubes (Biosphere SafeSeal Tubes 1.5 mL) and Endosafe pipet tips were purchased from Sarstedt (Nümbrecht, D). For the LAL test, 96-well flat bottom microtitre plates (M9005, endotoxin free, Endosafe, CRIVER) were used. For the multipette, combitips were purchased from Eppendorf (Combitips plus, Biopure; Hamburg, D).

### Single concentration sorption experiment

A 1000 EU/mL Endosafe control endotoxin standard was diluted in pyrogen free LAL water (LRW) to yield a working solution of 10 endotoxin Units per millilitre (EU/mL). The endotoxin activity in the stock solution was verified by the LAL test. Five milligram of PMB (positive control) and each sorbent were dissolved in 5 mL of the LPS working solution to yield a final concentration of 0.1% w/v sorbent. Reaction mixtures and pure sorbents were shaken at 112 × g for 2 hours on a micro plate shaker at room temperature (22 ± 1°C). Afterwards, the samples were centrifuged at 500 × g for 15 min, supernatant of reaction mixtures and pure sorbents were diluted in LRW water (1:100, 1:10) and endotoxin activity was measured using the LAL assay.

### Influence of endotoxin concentration on adsorption efficiency

We used a 250,000 EU/mL stock of LPS of *E. coli* O55:B5 from Sigma for preparing eight concentrations of endotoxin working solutions, ranging from 2,500-50,000 EU/mL. The endotoxin activity in the stock solution was verified by the LAL test. Each sorbent (5 mg) was dissolved in 5 mL of LRW solution to yield a concentration of 0.1% w/v of sorbent. Incubation was done shaking at 112 × g for 2 hours at 37°C. LPS working solutions and reaction mixtures were diluted and measured as described above. Reaction mixtures had to be diluted up to 1:10,000 before applying on the plate and measured using the LAL assay.

### Artificial intestinal fluid sorption experiment

The artificial intestinal fluid (AIF) was prepared according to the “Official Method – Determination of the Disintegration Time of Tablets” by Health Canada ( 1989). LPS working solutions were prepared from a ≥250,000 EU/mL stock of LPS of *E. coli* O55:B5 from Sigma to gain concentrations of 25,000 and 120,000 EU/mL. Five milligrams of sorbents and PMB were dissolved in 5 mL AIF to gain a 0.1% w/v solution. Samples were incubated shaking at 112 × g for 2 hours at 37°C. Thereafter, reaction mixtures were treated as described in paragraph “*Influence of endotoxin concentration on adsorption efficiency”.*

#### Limulus amebocyte lysate test (LAL)

The test was performed using a 96-well microtitre plate and optical density was read at 405 nm. Reaction was measured over 70 minutes. For each clay mineral, 100 μL of the diluted adsorbent alone and the sample LPS mixture was applied in duplicate onto the 96-well-plate. Spiking experiments were carried out in order to determine the LPS recovery for identifying interferences of endotoxins, reagent and used materials. For that purpose, a spiking solution with a concentration of 10 EU/mL was prepared. Subsequently, 50 μL of the spiking solution was added to the supernatant of the reaction mixtures. Each sample and the respective spiked sample were diluted to meet the calibration line and applied in duplicate. Preparation of a four-point calibration line (0.05, 0.5, 5, 50 EU/mL), application of 100 μL reaction reagent (Endochrome K as a chromogenic substrate) and the measurements were performed according to European Pharmacopoeia 5.0 2005 (chapter 2.6.14., Method D, pp. 161–168). For all experiments done in LRW solution, calibration line was done with LRW. For experiments in AIF solution, calibration line was assayed in AIF buffer. Data were calculated by the EndoScanV 9.1 software (CRIVER).

### Performance standards of the LAL test

Performance characteristics of the LAL test were defined according to manufacturer’s specifications: The LPS recovery of the spiked sample had to be 50% to 200%, and the coefficient of variation of the sample, analysed in duplicate, had to be lower than 10%. Additionally, the coefficient of correlation of the calibration line had to be equal or better than 0.980. Each plate included a negative control (endotoxin free test water, LRW) and the related spiked sample. Invalid recoveries of spiked samples show false negative or false positive readings therefore, they can be excluded. Data which did not fulfil the performance standards of the test were not considered in our calculations.

### Endotoxin removal efficiency (E)

The endotoxin removal efficiency was calculated for experiments in LRW and different increasing endotoxin concentrations and experiments in AIF by equation (1), where E was the removal efficiency in percentage (%), and C_0_ and C were the endotoxin concentrations measured in the stock solution and in sample supernatant, respectively.

(1) $$E=\left(\frac{C_{0}-C}{C_{0}}\right)\;*\;100$$

### Endotoxin adsorption capacity (q) and isothermal equations

In comparison to PMB, two sorbents were chosen to calculate the amount of endotoxin adsorbed (q) and to fit them to isothermal equations. Therefore, the endotoxin activity (EU/mL) was converted into endotoxin EU/mg sorbent. This referred to the potency of the used endotoxin standard, which was defined by the certificate of analysis from the producer, to be between 7–12 EU/ng (depending on the certificate of the standard/charge).

To calculate the adsorbed amount of endotoxin on the control and the used sorbents (q), the following equation (2) was used

(2) $$q=\left(\frac{C_{0}-C}{W_{m}}\right)\;\times \;V$$

C_0_ and C were the concentrations of endotoxin in the initial endotoxin solution and in the supernatant after adsorption. V was the volume of the solution (mL) and W_m_ was the amount of adsorbent used (mg).

The data were fitted to different isothermal models (linear and Freundlich isotherm). The equation used for the linear model (3) was

(3) $$q=K_{d}\;\times \;C$$

and the equation used for the Freundlich model (4) was

(4) $$q=K_{F}\;\times \;C^{\frac{1}{n}}$$

For both equations, q corresponded with the concentration of endotoxin bound (EU/mg). C was the concentration of endotoxin in solution (EU/mL). K_d_ represented the dimensionless distribution coefficient. K_F_ corresponded to the Freundlich coefficient. The Freundlich exponent n described the deviation of the isotherm from a linear correlation and was a measure for the sorption intension. If n = 1, a linear isotherm is described and the distribution between the two phases is independent of the concentration of the sorbent. 1/n < 1 is a reference for a normal Langmuir isotherm and 1/n > 1 indicates a cooperative sorption.

### Calculations and diagrams

All calculations for the isotherm models were done with Table Curve 2D v5.0. Diagrams and tables were prepared with Microsoft Excel 2010. For statistical comparison of mean binding efficiencies and to demonstrate significant decrease of endotoxin activity when sorbents used, a one-sided *T*-test (equal variances not assumed; Welch correction) was performed.

## Results

### Single concentration sorption experiment

Sorption of endotoxin with four different sorbents in comparison to PMB (positive control) was determined. The adsorption (%) of tested substances is presented in Table 1. The measured endotoxin activity of the used solution was 12.5 ± 2.7 EU/mL. Measurements were done in duplicate in two separated tests (1, 2).

**Table 1:** **Data represent the adsorption efficiency [%] for endotoxin in a 0.1**% **(w/v) sorbent solution**

**Sorbents**	**Test**	**PMB**	**Organoclay 1**	**Organoclay 2**	**Bentonite 1**	**Bentonite 2**
Percentage bound (%)	1	100	100	100	0	0
	2	100	100	100	14	12

The positive control PMB, along with Organoclay 1, bound 100% of the available endotoxin, whereas the products Bentonite 1 and Bentonite 2 showed a low binding efficiency.

### Influence of endotoxin concentration on adsorption efficiency

**Figure 1:**
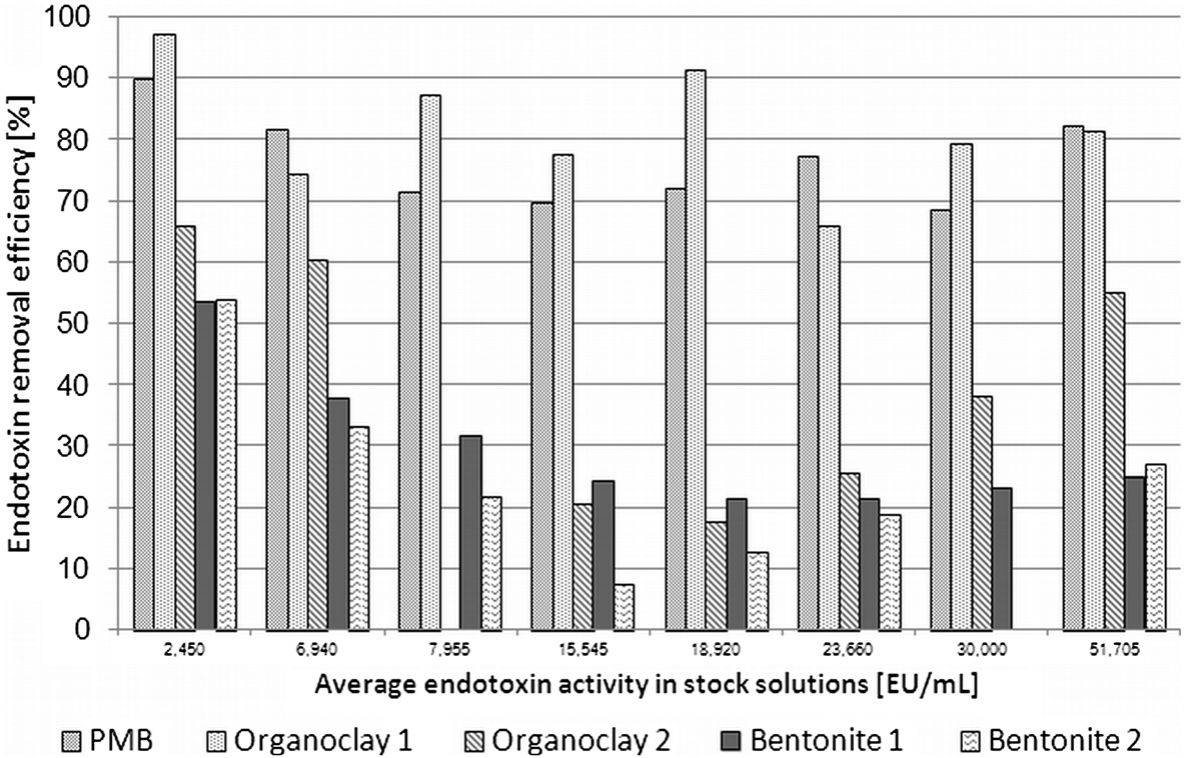
**Average endotoxin binding efficiencies (%) of different sorbents under given conditions (37°C , 2 h incubation) in different aqueous solution with different endotoxin concentrations.**

Endotoxin concentration studies on adsorption efficiency were performed at 37°C, over a 2 hour incubation period. Eight endotoxin concentrations were used and the measured activity ranged from 2,450 – 51,700 EU/mL aqueous solution. The binding efficiencies (E) of the given sorbents compared to the control are shown in Figure 1. Organoclay 1 showed a comparable (P = 0.385) binding efficiency (average 81.6%) than the positive control PMB (average 76.4%). The removal efficiency of Organoclay 2 varied strongly in dependence on the endotoxin concentration (min 18%, max 66%). The two non-organophilised products, Bentonite 1 and Bentonite 2, showed on average the lowest binding efficiencies ranging between 21 and 54% and 7 to 52%, respectively. Organoclay 2 (P = 0.013), Bentonite 1 (P = 0.026) and 2 (P = 0.022) showed a significantly decreased binding capacity compared to PMB.

**Figure 2:**
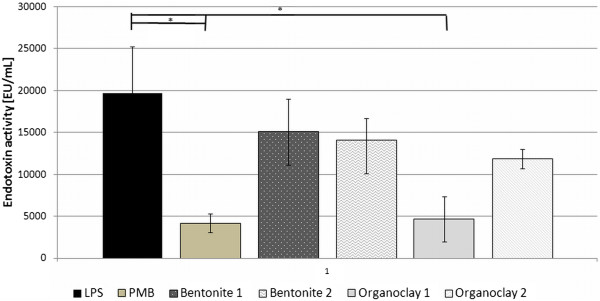
**Summary of mean endotoxin activities over all concentrations.** Error bars stand for standard error means. Asterisk indicate significant decrease (P < 0.05) of endotoxin activity of LPS versus used sorbents.

Figure 2 summarizes the mean measured endotoxin values over all tested concentrations. Only PMB (P = 0.015) and Organoclay 1 (P = 0.017) significantly decreased the endotoxin activity in LRW solutions. Bentonite 1 (P = 0.268), Bentonite 2 (P = 0.213) and Organoclay 2 (P = 0.119) revealed no significant lower endotoxin values.

**Figure 3:**
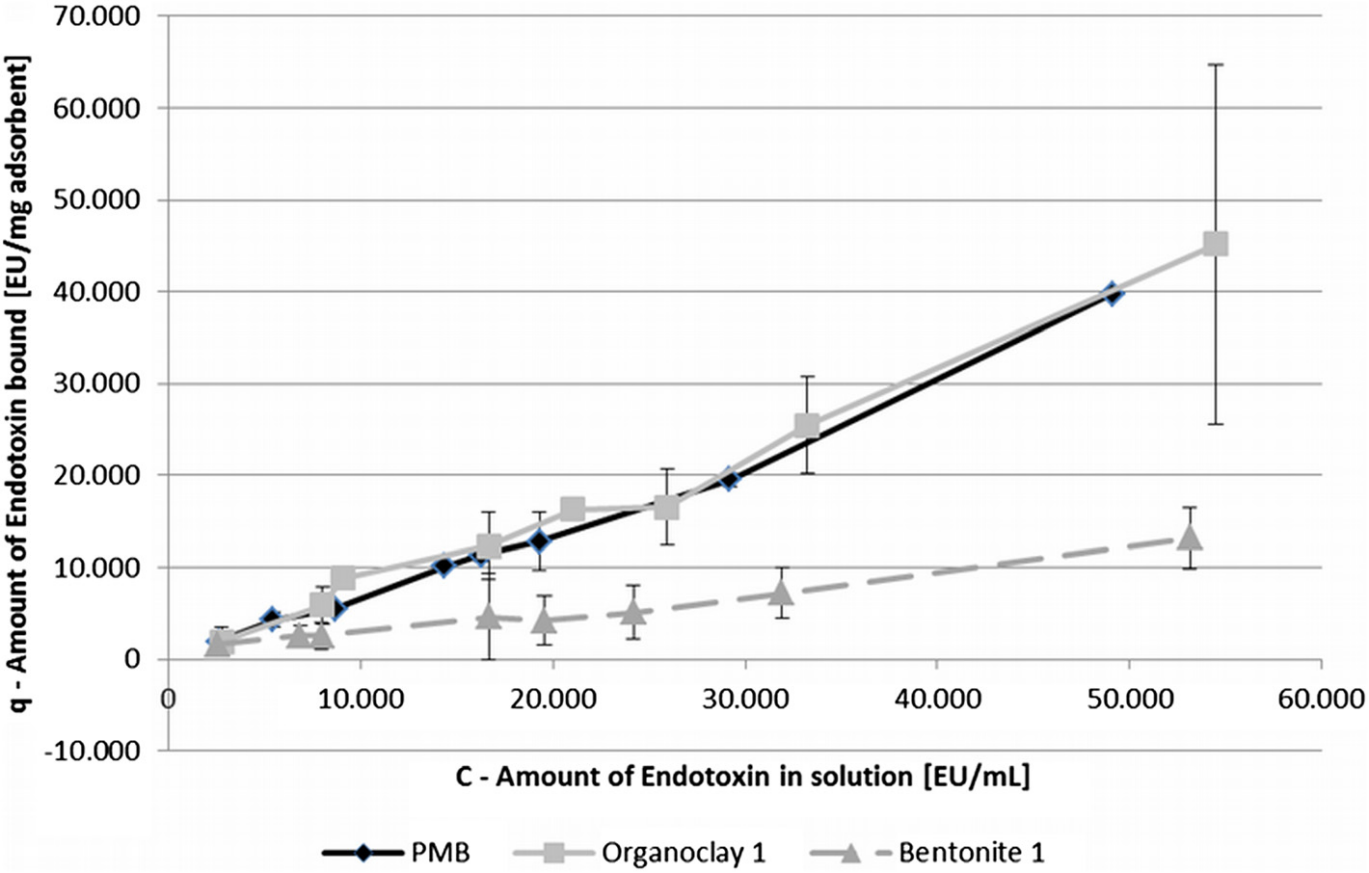
**The relationship between adsorption capacity (q) and amount of endotoxin in solution (C) of different sorbents.** Error bars represent the minimum and maximum measured amount of endotoxin bound within two independent replicates.

Further tests and calculations were only investigated with PMB, Organoclay 1 and Bentonite 1. On the one hand, as those sorbents showed constant results in previous tests and on the other hand, to compare a low and a high binding sorbent for their binding mechanism. To give an indication on the bound amount of endotoxin (q) in correlation to the initial endotoxin activity in aqueous solution (C), the correlation of two sorbents and PMB is shown exemplarily in Figure 3. The progression of the gradient of Organoclay 1 showed similarity to the control, suggesting a comparable binding efficiency. The progression of Bentonite 1 is similar to Organoclay 1 but shows a decreased amount of bound endotoxin. None of the used clays reached a saturation value and therefore, no maximum sorption coefficient could be calculated (q_max_, Langmuir isotherm).

Results were also expressed as sorption isotherms to identify the adsorption mechanism. For this experiment the linear and the Freundlich model were chosen. Table 2 presents results for K_d_, K_F_ and respective R and 1/n. Organoclay 1 and PMB showed a better coefficient of correlation with the Freundlich isotherm than with the linear model. Result of Organoclay 1 and PMB (1/n > 1) support the idea of a Langmuir isotherm. Langmuir isotherm could not be applied, as no equilibrium concentration for sorbents could be determined. Bentonite 1 revealed lower coefficients of correlation for both models and compared to the other sorbents 1/n = 1, which would suggests a linear isotherm.

**Table 2:** Parameters determined by fit to two models of the isotherm data for binding of sorbents

	**Linear model**		**Freundlich model**		
**Adsorbent**	**K**_**d**_	^**a**^**R**^**2**^	**K**_**F**_	**1/n**	^**a**^**R**^**2**^
PMB	0.75	0.98	0.15	0.87	0.99
Organoclay 1	0.79	0.98	0.28	0.92	0.99
Bentonite 1	0.24	0.96	0.36	1.04	0.96

^a^Coefficient of correlation.

### Artificial intestinal fluid (AIF) sorption experiment

**Figure 4:**
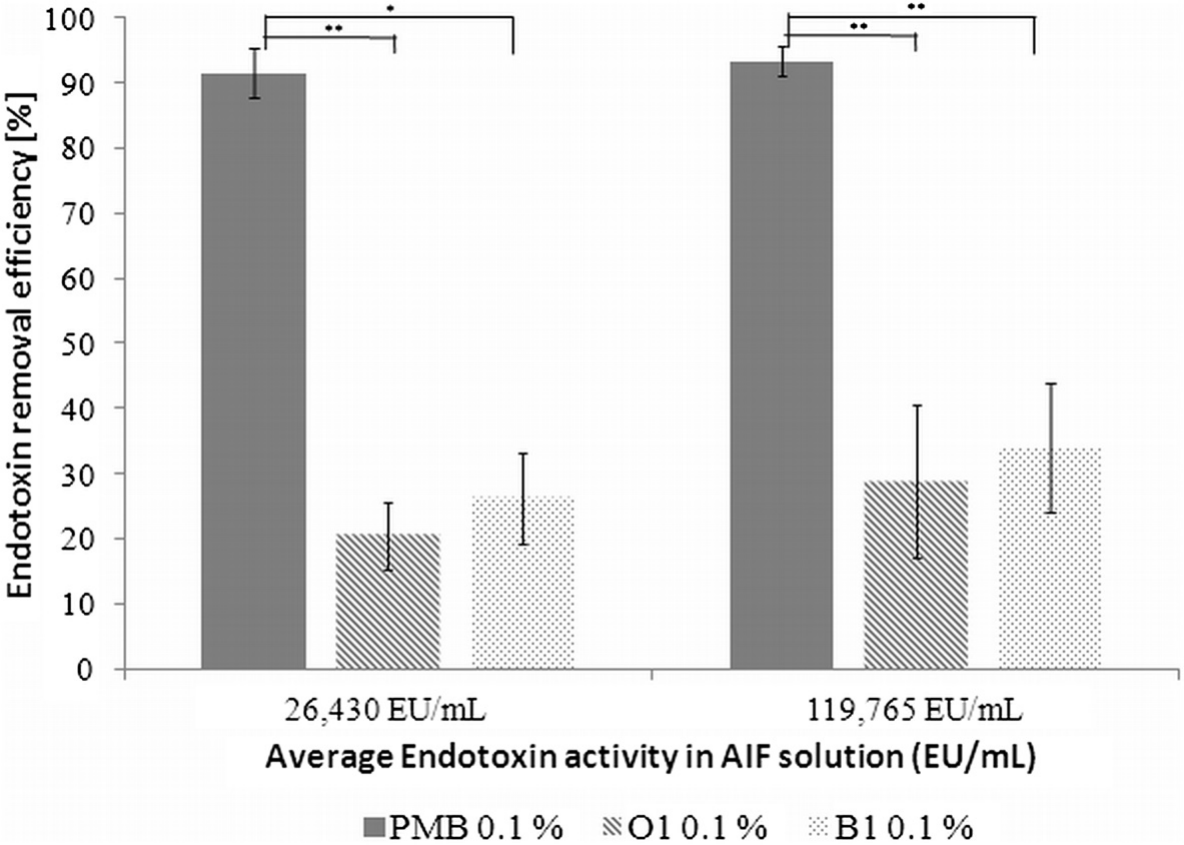
**Average of endotoxin binding efficiencies (%) of different sorbents under given conditions (37°C , 2 h incubation) in one endotoxin concentration in AIF.** Error bars represent min and max measured deviations of mean binding efficiency. O1 stands for Organoclay 1 and B1 for Bentonite 1. Asterisk show significant differences (P < 0.05) between PMB and used sorbents.

Binding experiments in AIF were performed at two different endotoxin concentrations using two sorbents and control in 0.1% (w/v) solution. The aim was to investigate the endotoxin binding efficiency of clay minerals in a complex medium. The pure AIF revealed no endotoxin activity itself (<0,005 EU/mL). Binding efficiency of PMB and the tested clays are demonstrated in Figure 4. Concentrations of the LPS-AIF solutions were measured to be 26,430 EU/mL and 119,765 EU/mL, respectively.

PMB bound more than 90% of the added endotoxin at both endotoxin concentration levels. Organoclay 1 and Bentonite 1 showed binding efficiencies below 50%, whereas, Organoclay 1 showed a decreased binding efficiency compared to Bentonite 1 at both endotoxin concentrations.

## Discussion

The use of clay minerals as a feed additive for farm animals to prevent the effects of different mycotoxins (e.g. aflatoxin and ergotamine), bacteria and other toxic compounds is widespread and has been discussed for years (Slamova *et al.*^2011^; Tateo and Summa 2007; Trckova *et al.*^2004^). Therefore, the hypothesis is that binding endotoxins in the gut lumen will reduce the number of endotoxins entering the organism. As animal studies are expensive, and the effect of the additive has to be assured, the *in vitro* screening of sorbents is important for estimating their adsorptive potential. Although, results of *in vitro* studies may not be reflected in *in vivo* studies, conclusions regarding the mode of action under controlled conditions can be gained (Ganner and Schatzmayr 2012). Approaches for screening binding materials *in vitro* and collecting information on the underlying functional mechanisms, are of great interest.

The role of endotoxins in inflammatory processes induced by Gram-negative bacteria is well known (Rietschel *et al.*^1994^). Already small amounts of endotoxin (< 1 ng/mL; approximately 10 EU/mL) (Anspach and Hilbeck 1995; Mani *et al.*^2012^) given intravenously are sufficient to activate the immune system and trigger symptoms (Gorbet and Sefton 2005). Even far higher dosages are expected to be present in the environment of farm animals (Ratzinger 2010; Vogelzang *et al.*^1998^).

PMB is a well investigated polypeptide antibiotic, which deactivates LPS via disrupting the molecular structure (Pristovsek and Kidric 1999). A two way mechanism explains the reaction. Firstly, the positively charged PMB adheres to LPS lamella because of electrostatic interaction and secondly, the acyl chain is inserted in the LPS lamella. Due to this stoichiometric binding effect, PMB was chosen as positive control in our experiments. The limitation of *in vivo* use of PMB is its toxicity (Danner *et al.*^1989^) and therefore, alternatives are necessary.

In our experiments, non-treated and organophilised bentonites were tested to get an idea of the endotoxin binding behaviour of sorbents in different media, and whether the LAL assay can be used to quantify endotoxin activity in different media. It has to be kept in mind that the use of organoclays as feed additives is not permitted in the European Union, but since organoclays are known to bind a broad spectrum of substances they were included in this study. In contrast, Bentonite-montmorillonite is authorized as a technological feed additive according to European Parliament regulation No 1831/2003 and can therefore, be used as a feed additive.

In older literature, fluorescein isothiocyanate (FITC)-LPS was used for fluorescence measurements (Nolan *et al.*^1975^) or the gel tube test was used to obtain quantitative results to measure the endotoxin binding *in vitro* (Ditter *et al.*^1983^). Gorbet and Sefton ( 2005) stated that many studies on materials incubated in endotoxin solutions were never reported. This fact points out the necessity of defined approaches for testing potential endotoxin sorbents.

 (Our investigations were based on the set-up of testing aflatoxin binding by Lemke *et al.* ( 2001). Our first approach was a single sorption experiment with a low amount (about 10 EU/mL) of endotoxin. Differences in adsorption efficiencies of organophilic and non-treated clays were revealed. The reason that organoclays bind endotoxin to a high percentage in a clear buffers solution may be explained by the hydrophobic forces between LPS and treated clays (Hou and Zaniewski 1991). Another explanation could be that, low endotoxin values in aqueous solution may not activate the binding sites of untreated bentonite.

However, far higher dosages than 10 EU/mL are expected in the environment. Thus, the next step in our experiments was, to test the influence of endotoxin concentrations (2,450 – 51,700 EU/mL in LRW solution) on the adsorption efficiency of sorbents. Organoclay 1 revealed on average a comparable binding efficiency to PMB (P = 0.385). The same behaviour was expected for Organoclay 2, however, binding efficiencies were significantly (P = 0.013) decreased compared to PMB. This could be either due to the differences of the original clay (before treatment) itself or to the treatment with different surfactants. In contrast to the swelling bentonites, which partially disperse themselves spontaneously in water owing to the swelling caused by penetration of water between the unit layers, the three-dimensional structure of attapulgite prohibits any internal swelling action, which reduces access to adsorbed molecules.

 (Further calculations with isothermal models were conducted. Data of PMB, Organoclay 1 and Bentonite 1 were fitted to linear and Freundlich isotherm according to the method explained by Giles *et al.* ( 1974). PMB and Organoclay 1 revealed comparable results and suggest a Langmuir isotherm, whereas Bentonite 1 indicated a linear isotherm. No saturation levels were reached for tested sorbents, although high concentrations of endotoxin were used.

In anticipation that the material might be used as feed additive, we implemented binding experiments in artificial intestinal fluid. Samples prepared with the lower concentrated endotoxin solution showed a decreased binding efficiency of sorbents compared to higher concentrated LPS test solutions. This observation can have two reasons: Firstly, toxins bind to sites with higher affinity, followed by binding to lower affinity sites, when toxin concentration increases (Ferraz *et al.*^2004^). Secondly, increased toxin concentrations change the surface of the sorbent and activate further binding sites (Giles *et al.*^1974^) or start binding previously bound molecules (Grant *et al.*^1998^).

When compared to binding results in LRW solutions with 23,660-30,000 EU/mL (around 72%), Organoclay 1 showed a significant decrease in binding efficiency in AIF (20%; P < 0.005). In comparison to these results, binding efficiencies in LRW solution (22%) of Bentonite 1 are comparable to results in AIF solution (26%). Thus, complex media can have an influence on binding capacity of clay minerals, in our case Organoclay 1 showed a decrease of binding efficiency of over 50%. This is a very important observation, since the binding agent should act in the gastrointestinal tract, where conditions are rather complex.

These results lead to the conclusion that the adsorption of endotoxins to organoclays is mostly unspecific which limits its practical application. In addition to that organoclays do not meet regulatory requirements for feed additives.

Overall, there are only a few references on *in vitro* endotoxin binding studies which were done using the kinetic chromogenic LAL test, and most of these studies were done with other sorbents than clay minerals. Anspach and Hilbeck ( 1995) investigated various affinity sorbents (histidine, histamine and PMB) and recovered a binding capacity of 0.48 mg endotoxin/g wet sorbent in a working solution with 70,000 EU/mL (7 μg/mL). In a master’s thesis from Xu Ying ( 2006) the *E. coli* and endotoxin adsorption capacity of montmorillonite, zeolite and carbon nanotube with and without cetylpyridinium chloride (quaternary ammonium compound) treatment was described. Maximum binding capacity was reached by treated carbon nanotubes (with 357 EU/mg sorbent ≈ 35.7 ng/mg) and lowest was with zeolite (76.8 EU/mg ≈ 7.68 ng/mg). All tests were performed in 10 EU/mL working solution. Whereas, Perianayagam and Jaber ( 2008) tested Sevelamer Hydrochloride (a nonadsorbable, cross-linked polymer) in a 100 EU/mL solution and found a concentration dependent effect with best results (96%) at 50 mg/mL Sevelamer Hydrochloride. In a study testing bone char, Rezaee *et al.* ( 2009) revealed a binding capacity of 2.9 ng Endotoxin/g sorbent in a working solution with 80 EU/mL. With regard to a different experimental set up, to the studies mentioned above, in aqueous solution we could calculate the following absorbent capacities for the highest endotoxin concentration (50,000 EU/mL): 5.59 mg/g (Organoclay 1) > 3.97 mg/g (PMB) > 2.58.mg/g (Organoclay 2) > 1.55 mg/g (Bentonite 1) > 1.23 mg/g (Bentonite 2). Even higher maximum binding capacities of sorbents may be expected as saturation was not reached in our studies. In general we could achieve higher binding efficiencies in our experiments compared to other studies.

In conclusion, the LAL assay could be successfully used to test supernatants of endotoxin binding studies in aqueous solution and artificial intestinal fluid. The studies we evaluated (single concentration, endotoxin concentrations on adsorption efficiency in LRW solutions and sorption experiments in AIF) provide a good tool for the screening of sorbents for their potential endotoxin binding efficiency. Our results show that incubation media used for the endotoxin binding assay have an influence on the binding capacity of clay minerals. Binding mechanisms of treated and non-treated clays are different. Organoclay 1 (0.1%) showed good binding efficiencies in LRW solutions, whereas Bentonite 1 (0.1%) obtained a lower, but constant binding efficiency of high concentrated LPS solutions in LRW and AIF. In the 50,000 EU/ml aqueous buffer solution best adsorbent capacity was revealed for Organoclay 1 (5.59 mg/g), which showed a comparable binding efficiency to PMB (3.97 mg/g). Even higher binding capacities may be approached if saturation would be reached. Clay minerals, non-treated and treated, showed a promising endotoxin binding capacity *in vitro*. Further investigations are necessary to better understand the binding mechanism and to prove the effect of the used clays for *in vivo* trials.

## Competing interests

The authors declare that they have no competing interests.
